# In Vitro Antimicrobial Activity of Ceftobiprole and Comparator Agents Against *Streptococcus pneumoniae* Responsible for Lower Respiratory Tract Infections in the United States (2016–2020), Including Resistant Subsets and Select Serotypes

**DOI:** 10.3390/antibiotics15040375

**Published:** 2026-04-07

**Authors:** Helio S. Sader, Mariana Castanheira, Mark E. Jones, Rodrigo E. Mendes

**Affiliations:** 1 Element Iowa City (JMI Laboratories), North Liberty, IA 52317, USA; 2 Basilea Pharmaceutica International Ltd., 4123 Allschwil, Switzerland

**Keywords:** CABP, ceftriaxone, PCV, pneumonia, respiratory infection

## Abstract

**Background**: Ceftobiprole is an advanced-generation cephalosporin approved in Europe in 2013 for various indications, and in the United States (US) in 2024 for community-acquired bacterial pneumonia (CABP), acute bacterial skin and skin structure infections, and *Staphylococcus aureus* bacteremia, including right-sided endocarditis. **Methods**: The *in vitro* activity of ceftobiprole and comparators was evaluated against 2793 *Streptococcus pneumoniae* causing lower respiratory tract infections in 32 US sites (2016–2020), including against subsets from various geographic regions, resistance phenotypes and prevalent serotypes. **Results**: Ceftobiprole inhibited 99.5% of all *S. pneumoniae* at the MIC of ≤0.5 mg/L (MIC_50/90_, 0.015/0.25 mg/L). Susceptibilities of 98.2% to 100% were observed for ceftobiprole against isolates originating from each surveyed year or each US Census Division. Ceftobiprole retained activity against isolates resistant to macrolides (98.8%), tetracycline (98.2%), oral penicillin (95.4%), against multidrug-resistant isolates (97.0%), and various serotypes (93.8–100%). Ceftriaxone (97.4%) and amoxicillin–clavulanate (95.1%) also showed elevated susceptibilities overall, but inconsistent results and lower than those observed for ceftobiprole were noted against isolates with elevated penicillin MIC or specific serotypes (i.e., 19A). **Conclusions**: These *in vitro* results, coupled with documented clinical efficacy, indicate that ceftobiprole is a valuable option to treat CABP caused by *S. pneumoniae* in the US.

## 1. Introduction

*Streptococcus pneumoniae* is an important human pathogen and is responsible for various types of infections, including community-acquired bacterial pneumonia (CABP), meningitis, bacteremia, sepsis, otitis media, and sinusitis [1]. The treatment of CABP caused by *S. pneumoniae* continues to rely on empirical antibiotic therapy, and antibiotic selection is usually based on local/regional antimicrobial susceptibility patterns and an assessment of risk factors for antimicrobial resistance, which often varies nationally and regionally [2]. Guidelines for empirical treatment recommend that when a certain threshold of antimicrobial resistance is reached, choices of antibiotic therapy should change accordingly [3]. In addition, it is also important to consider differences in the antimicrobial susceptibility profile of isolates causing invasive versus non-invasive infections [4,5].

An increased use of pneumococcal conjugate vaccines (PCVs) will probably lead to a reduction in the prevalence of antimicrobial-resistant vaccine-type *S. pneumoniae* and will indirectly improve the activity of antimicrobial agents generally used for the treatment of pneumococcal infections against the overall population and vaccine-type isolates. However, antimicrobial resistance may continue to persist and/or increase in certain locations by various mechanisms, including misuse of antimicrobial agents, the dissemination of clones with evolutionary advantages, and serotype replacement, where the serotypes targeted by the vaccine are replaced by non-vaccine type antimicrobial-resistant *S. pneumoniae* [6]. Thus, it is important to maintain continuous monitoring of serotypes causing invasive and non-invasive infections, and the antimicrobial susceptibility profile of *S. pneumoniae* [7].

The American Thoracic Society (ATS)/Infectious Diseases Society of America (IDSA) guideline for the management of CABP recommends β-lactam, macrolide, doxycycline, or a respiratory quinolone monotherapy for outpatients with non-severe infections. Combination therapy of a β-lactam and a macrolide or tetracycline (i.e., doxycycline), or monotherapy with a respiratory quinolone is recommended for patients with comorbidities [8]. However, resistance to these agents may represent a clinical challenge to the guided treatment, as well as to the empirical treatment, because resistance rates to many options are beyond the threshold recommended by the guidelines [1,2].

Resistance to β-lactams in *S. pneumoniae* is primarily due to mutations in the *pbp* genes, which lead to reduced susceptibility to penicillins, oral cephalosporins, and ceftriaxone [1]. However, some advanced cephalosporins, such as ceftobiprole and ceftaroline, retain high binding affinity to the modified PBPs responsible for resistance in *S. pneumoniae* [9,10]. Ceftobiprole is a fifth-generation cephalosporin, which has shown non-inferiority to ceftriaxone in a double-blinded, multicenter, randomized trial with linezolid in cases of high risk of ceftriaxone-resistant *S. pneumoniae* or methicillin-resistant *Staphylococcus aureus* (MRSA) in CABP [9]. Ceftobiprole has also shown non-inferiority to ceftazidime plus linezolid in a double-blind, multicenter, randomized study in HAP [10]. Ceftobiprole exhibits bactericidal activity against Gram-positive bacteria, including penicillin-resistant *S. pneumoniae*, MRSA, non-ESBL-producing Enterobacterales, and some *Pseudomonas aeruginosa* isolates. Ceftobiprole activity against penicillin-resistant *S. pneumoniae* and MRSA is due to its strong binding to altered penicillin-binding proteins (PBPs), such as PBP1a, PBP2x, and PBP2b from penicillin-resistant *S. pneumoniae* and PBP2A from MRSA strains [11,12].

Ceftobiprole was approved by the European Medicines Agency (EMA) in 2013 to treat nosocomial pneumonia, complicated skin and skin structure infections, and diabetic foot infections [13] (https://www.basilea.com/news/news?tx_news_pi1%5Baction%5D=detail&tx_news_pi1%5Bcontroller%5D=News&tx_news_pi1%5Bnews%5D=493&type=1546938654&cHash=02d70b683af21d8251ef6b38f5195806 [accessed on 18 February 2026]). Ceftobiprole has also been marketed in Canada and Switzerland to treat complicated skin and skin structure infections, including minor diabetic foot infections without osteomyelitis, for many years. In April 2024, ceftobiprole was approved by the United States (US) Food and Drug Administration (FDA) for the treatment of CABP, acute bacterial skin and skin structure infections, and *S. aureus* bacteremia, including right-sided endocarditis (https://www.fda.gov/news-events/press-announcements/fda-approves-new-antibiotic-three-different-uses [accessed on 18 February 2026]) [11,12]. This study evaluated the *in vitro* antimicrobial activity of ceftobiprole and comparator agents against *S. pneumoniae* isolates, including resistant subsets and prevalent serotypes, causing community-acquired respiratory tract infections in patients from US medical centers during 2016–2020.

## 2. Results

Overall, ceftobiprole (MIC_50/90_, 0.015/0.25 mg/L) was active against 99.5% of the entire *S. pneumoniae* population at the US FDA susceptibility breakpoint of ≤0.5 mg/L (Table 1). Notably, ceftobiprole retained activity against 79.2% of ceftriaxone-nonsusceptible isolates (*n* = 72; MIC_50/90_, 0.5/1 mg/L). All ceftobiprole nonsusceptible isolates were also not susceptible to ceftriaxone (5 isolates were ceftriaxone-intermediate [MIC of 2 mg/L] and 10 were ceftriaxone-resistant [MIC > 2 mg/L]), and resistant to oral penicillin (MIC ≥ 2 mg/L). Only two isolates were categorized as ceftobiprole-resistant according to US FDA criteria (MIC ≥ 2 mg/L), both *S. pneumoniae* isolates with an MIC of 2 mg/L (Table 1). Ceftobiprole activity was also elevated against isolates originating from each surveyed year or each US Census Bureau Division, with susceptibility rates varying from 98.2% to 100%. In general, 97.4% and 95.1% of all *S. pneumoniae* isolates were susceptible to ceftriaxone and amoxicillin–clavulanate, respectively, whose activities (95.7–99.2% for the former and 92.5–98.8% for the latter) were also elevated against isolates from each year or US Census regions (Table 2).

**Figure 1:**
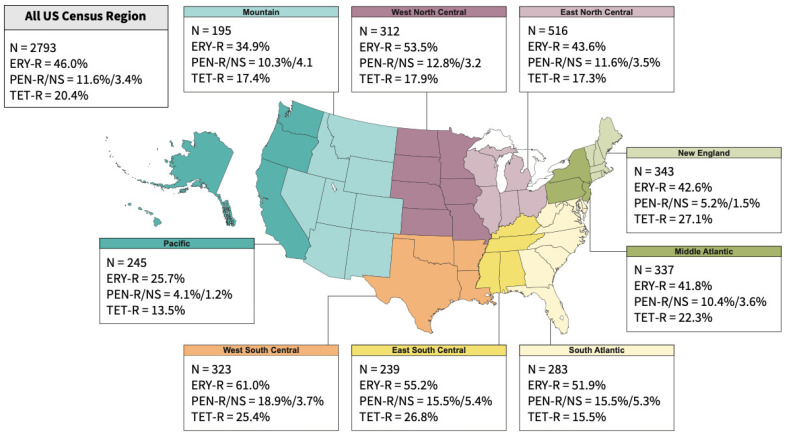
Number of isolates included in the study and percentage of erythromycin-resistant (ERY-R), oral/parenteral penicillin-resistant/nonsusceptible (PEN-R/NS) and tetracycline-resistant (TET-R) overall and by US Census Bureau Division.

Susceptibility rates lower than 90% were observed for clindamycin (85.5%), erythromycin (53.2%), oral penicillin (63.2%), tetracycline (79.4%), and TMP-SMX (72.8%) (Table 2). The susceptibilities for these comparator agents varied broadly among US Census regions (Table 2), and the resistance and nonsusceptibility rates for select comparators are shown in Figure 1. When ceftobiprole activity was evaluated against isolates resistant to these comparator agents, ceftobiprole retained activity and had MIC_90_ values of 0.5 mg/L and susceptibilities of 95.4–98.8% against 386 isolates resistant to clindamycin (MIC ≥ 1 mg/L), 1286 isolates resistant to erythromycin (MIC ≥ 1 mg/L), 325 isolates resistant to oral penicillin (MIC ≥ 2 mg/L), 570 isolates resistant to tetracycline (MIC ≥ 2 mg/L), 453 isolates resistant to TMP-SMX (MIC ≥ 4 mg/L), and 505 isolates showing a MDR phenotype (Table 1 and Table 2). Among a select group of 96 isolates with MIC values ≥ 4 mg/L for penicillin (nonsusceptible to parenteral penicillin), 84.4% of the isolates were susceptible to ceftobiprole. In contrast, the comparator agents, ceftriaxone (78.5–89.3% susceptible) and amoxicillin–clavulanate (58.6–89.6% susceptible), were active against less than 90% of these resistant subsets, except for ceftriaxone against erythromycin-resistant isolates, where 94.4% of the isolates were inhibited at the ceftriaxone-susceptible breakpoint (Table 2).

The activity of ceftobiprole, ceftriaxone and other comparator agents was also evaluated against a subset of 625 isolates with serotyping information available, more specifically, against the most common serotypes containing >25 isolates. In general, the activities of ceftobiprole (100% susceptible), ceftriaxone (98.7–100% susceptible), and amoxicillin–clavulanate (94.7–100% susceptible) were elevated against each of the most prevalent serotypes, except against serotype 19A, where ceftobiprole remained active against 93.8% of these isolates, whereas ceftriaxone and amoxicillin–clavulanate inhibited only 62.5% and 18.8% of these isolates at their respective susceptibility breakpoints (Table 2). The susceptibilities of clindamycin, erythromycin, oral penicillin, tetracycline and TMP-SMX varied markedly according to serotype. In contrast, all isolates were susceptible to linezolid, vancomycin, and 96.9% to 100% of isolates were also susceptible to levofloxacin, regardless of year surveyed, region, resistance phenotype, or serotype; however, a lower susceptibility (87.9%) was observed for levofloxacin against the serogroup 22A/22F (Table 2).

## 3. Discussion

Surveillance programs provide important information for the generation of guidelines and for healthcare practitioners when treating bacterial respiratory infections. The SENTRY program has been monitoring the *in vitro* activity of ceftobiprole and other antimicrobials against clinically relevant bacterial pathogens, including *S. pneumoniae,* collected from episodes of respiratory tract infections, healthcare-associated pneumonia, bloodstream infections, and other infection types [14,15,16]. In this present investigation, ceftobiprole exhibited potent activity against a large collection of *S. pneumoniae* from US medical centers, including isolates resistant or nonsusceptible to the agents recommended for the treatment of CABP, such as β-lactams, erythromycin and tetracycline (doxycycline representative), and also isolates with a MDR phenotype.

Resistance to β-lactam antibiotics in *S*. *pneumoniae* is caused by consecutive alterations in the penicillin-binding domains of the PBPs, resulting from point mutations or mosaic genes [17]. These mutations may produce conformational changes in a loop that is adjacent to the entrance of the active-site cavity, obstructing β-lactam binding. Modified PBP 1a, PBP 2x, and PBP 2b are the most relevant PBPs related to β-lactam resistance among clinical isolates. These modified PBPs have low affinity for penicillins and cephalosporins, resulting in elevated MIC. In contrast, ceftobiprole still appeared to bind effectively to these altered PBPs, and remained active against subsets of isolates with elevated penicillin MIC values or serotypes (e.g., 19F) showing decreased susceptibility to penicillin, amoxicillin–clavulanate, and ceftriaxone [17,18,19]. Mohanty et al. evaluated antimicrobial resistance trends for *S. pneumoniae* isolates from adults with pneumococcal disease in the United States from 2011 to 2020. A total of 34,039 isolates were analyzed over the study period, and the investigators observed high rates of resistance to macrolides (37.7%), penicillin (22.1%), and tetracyclines (16.1%). Moreover, multivariate modeling identified a significant increasing trend in resistance to macrolides (+1.8%/year; *p* < 0.001) and decreasing trends for penicillin (−1.6%/year; *p* < 0.001), extended-spectrum cephalosporins (−0.35%/year; *p* < 0.001), and ≥3 drugs (−0.5%/year; *p* < 0.001) [20].

Ceftobiprole activity against *S. pneumoniae* with decreased susceptibility to other β-lactams has been reported by other investigators. The Canadian Ward (CANWARD) surveillance study evaluated the activity of ceftobiprole against >20,000 bacterial isolates from 16 medical centers in Canada, including 40 *S. pneumoniae* resistant to oral penicillin (MIC ≥ 2 mg/L). Ceftobiprole MIC_50_ and MIC_90_ values were 0.5 mg/L for both, and all isolates were inhibited at the US FDA susceptibility breakpoint of ≤0.5 mg/L [21]. Canton et al. evaluated the ceftobiprole susceptibility profiles against 20,000 bacterial isolates collected in 17 European countries in 2016–2019. Ceftobiprole MIC_50_ and MIC_90_ values against 148 *S. pneumoniae* resistant to oral penicillin were 0.5 and 1 mg/L, respectively, and 69.6% of isolates were susceptible to ceftobiprole [22]. Hawser et al. assessed ceftobiprole *in vitro* activity against 686 *S. pneumoniae* isolates from 16 European countries in 2019. Ceftobiprole activity was not presented according to penicillin susceptibility, but the ceftobiprole MIC_90_ value against the overall population was 0.5 mg/L (MIC_90_, 2 mg/L for penicillin), and 98.4% of isolates were susceptible to ceftobiprole at ≤0.5 mg/L, whereas 72.2% were inhibited at a penicillin MIC of ≤2 mg/L (parenteral susceptibility breakpoint) [23].

This study has some limitations. The fact that the isolates were collected in 2016–2020, and we do not have ceftobiprole susceptibility data after 2020, represents an important limitation. Although ceftobiprole was only approved for clinical use in the United States in April 2024, clinical use of other β-lactams (especially ceftaroline), the COVID-19 pandemic, and changes in serotype distribution could have affected the activity of ceftobiprole against *S. pneumoniae* since 2020. The limited number of isolates with serotype information available for analysis is another limitation of this investigation. A follow-up study describing the serotype distribution among *S. pneumoniae* isolates from the US included in the SENTRY program after the COVID-19 pandemic (2022–2023) reported subtle differences in individual serotypes when compared to the results presented here [7]. Additional studies also reported on the effect of COVID-19 on the incidence of individual serotypes [24]. Moreover, the elevated proportion (37.5%) of 19A isolates nonsusceptible to ceftriaxone remains a clinical concern. A previous study also reporting on 19A isolates from the SENTRY program causing pneumonia in patients hospitalized in US sites during 2009–2017 described proportions of nonsusceptibility to ceftriaxone varying from 24% to 64% [25]. These data suggest that the epidemiology of *S. pneumoniae* constantly evolves, and 19A isolates remained a significant burden in the USA despite the successful immunization programs [24]. Therefore, current surveillance data describing the ceftobiprole activity against a recent collection of *S. pneumoniae* is crucial, given the constant changes in the serotype distribution landscape and epidemiology. In summary, the results presented here corroborate previous publications on the *in vitro* activity of ceftobiprole and indicate that this compound exhibited potent *in vitro* activity against *S. pneumoniae* causing respiratory infections in the US, with consistent activity, regardless of surveyed year, geographic region, resistance phenotype and serotype/serogroup.

## 4. Materials and Methods

### 4.1. Clinical Isolates

A total of 2793 *S. pneumoniae* isolates were consecutively collected from patients with lower respiratory tract infections in 32 US medical centers distributed across all 9 US Census Bureau Divisions via the SENTRY Antimicrobial Surveillance Program for 2016–2020. All participant centers follow a unique protocol, which states that only isolates considered clinically relevant and only isolates per patient infection episode should be submitted to the central monitoring laboratory (Element Iowa City [JMI Laboratories], North Liberty, IA, USA) for confirmation of bacterial identification and susceptibility testing by the broth microdilution method. Isolates were mainly from good-quality sputum samples (with >25 polymorphonuclear leukocytes and <10 squamous epithelial cells per low-power field; 28.4%), tracheal aspirate samples (16.4%), sinus cavity samples (15.1%), bronchoalveolar lavage (14.8%), and blood culture (5.3%). Species identification was confirmed by standard biochemical tests and using the MALDI Biotyper (Bruker Daltonics, Billerica, MA, USA) according to the manufacturer’s instructions, where necessary.

### 4.2. Antimicrobial Susceptibility Testing and Serotyping

Broth microdilution was performed according to CLSI methods [26]. *S. pneumoniae* isolates were tested in cation-adjusted Mueller–Hinton broth (Becton and Dickson Company [BD]; Franklin Lakes, NJ, USA) supplemented with 2.5 to 5% lysed horse blood (Hemostat Laboratories; Dixon, CA, USA). Ceftobiprole powder was provided by Basilea Pharmaceutica International Ltd. (Allschwil, Switzerland), ceftaroline was obtained from Pantheon Pharma Services/Thermo Fisher Scientific (Waltham, MA, USA), and comparator powders were obtained from United States Pharmacopeia (USP; Rockville, MD, USA) or Sigma-Aldrich (Saint Louis, MO, USA). The quality control (QC) strain *S. pneumoniae* ATCC 49619 was tested concurrently with clinical isolates. Susceptibility determinations and quality assurance of MIC results were based on CLSI guidelines [26]. US FDA breakpoint criteria published in 2025 were applied for ceftobiprole (susceptible at ≤0.5 mg/L, intermediate at 1 mg/L, and resistant at ≥2 mg/L) [12]. CLSI criteria were applied for the comparator agents when available [27]. A total of 54.1% (*n* = 625) *S. pneumoniae* collected during 2016 and 2017 had serotyping information available, which was used in this analysis. The serotype information was obtained as previously described [25].

### 4.3. Data Analysis

In addition to the analysis based on the serotype information available, isolates were grouped according to resistance phenotypes using CLSI breakpoints [26]. Furthermore, isolates were clustered based on a multidrug resistance (MDR) phenotype, which was defined when nonsusceptibility was observed to 3 or more of the following antimicrobial agents: parenteral penicillin (MIC, ≥4 mg/L), ceftriaxone (MIC, ≥2 mg/L), erythromycin (MIC, ≥0.5 mg/L), clindamycin (MIC, ≥0.5 mg/L), levofloxacin (MIC, ≥4 mg/L), tetracycline (MIC, ≥2 mg/L), and trimethoprim–sulfamethoxazole (TMP-SMX) (MIC, ≥1/19 mg/L) [28].

## 5. Conclusions

Our results highlight the consistency of ceftobiprole activity across regions and resistant phenotypes. These results, coupled with documented clinical efficacy of ceftobiprole for the treatment of CABP [8,9], indicate that ceftobiprole is a valuable option for the management of respiratory infections caused by *S. pneumoniae*, especially when treating infections caused by isolates that may be refractory to penicillins and early-generation cephalosporins.

## Figures and Tables

**Table 1:** MIC distributions of ceftobiprole and cumulative percentages when tested against *S. pneumoniae* isolates (2016–2020).

Resistant Subset (No.) ^a^	Number and Cumulative % of Isolates Inhibited at MIC (mg/L):											MIC_50_	MIC_90_
	≤0.004	0.008	0.015	0.03	0.06	0.12	0.25	0.5	1	2	>2		
All Isolates (2793)	99	791	904	109	123	160	338	254	13	2		0.015	0.25
	3.5	31.9	64.2	68.1	72.5	78.3	90.4	99.5 ^b^	99.9	100			
Ceftriaxone-nonsusceptible (72)						1	1	55	13	2		0.5	1
						1.4	2.8	79.2	97.2	100.0			
Clindamycin-resistant (386)	4	27	54	20	29	71	70	101	10			0.12	0.5
	1.0	8.0	22.0	27.2	34.7	53.1	71.2	97.4	100				
Erythromycin-resistant (1286)	16	166	273	47	94	140	299	236	13	2		0.12	0.5
	1.2	14.2	35.4	39.0	46.3	57.2	80.5	98.8	99.8	100			
Levofloxacin-nonsusceptible (18)	1	5	4	0	2	2	3	1				0.015	0.25
	5.6	33.3	55.6	55.6	66.7	77.8	94.4	100.0					
Oral penicillin-resistant (MIC ≥ 2 mg/L; 325)						5	93	212	13	2		0.5	0.5
						1.5	30.2	95.4	99.4	100			
Parenteral penicillin-nonsusceptible (MIC ≥ 4 mg/L; 96)						1	3	77	13	2			
						1.0	4.2	84.4	97.9	100		0.5	1
Tetracycline-resistant (MIC ≥ 4 mg/L; 570)	15	61	60	27	84	106	88	119	10			0.12	0.5
	2.6	13.3	23.9	28.6	43.3	61.9	77.4	98.2	100				
TMP-SMX-resistant (453)	4	14	43	14	24	26	138	179	9	2		0.25	0.5
	0.9	4.0	13.5	16.6	21.9	27.6	58.1	97.6	99.6	100			
MDR (505)	5	22	53	23	80	101	83	123	13	2		0.12	0.5
	1.0	5.3	15.8	20.4	36.2	56.2	72.7	97.0	99.6	100			

Abbreviations: TMP-SMX, trimethoprim–sulfamethoxazole; MDR, multidrug-resistant. ^a^ Resistant isolates selected according to CLSI criteria [13]. Isolates nonsusceptible to parenteral penicillin were grouped instead due to the small number (11) of resistant isolates. ^b^ Underlined values represent percentage susceptible per the US FDA breakpoint [13].

**Table 2:** Susceptibility profiles of *S. pneumoniae* isolates from USA medical centers by year, US Census Divisions, resistance phenotype and serotype.

Isolates (No. Tested)			Antimicrobial Agent (% Susceptible) ^a^								
	BPR	CRO	AMC	CLI	ERY	LEV	LZD	PEN	TET	TMP-SMX	VAN
All (2793)	99.5	97.4	95.1	85.5	53.2	99.4	100	63.2/96.6	79.4	72.8	100
Year											
2016 (599)	99.7	97.5	94.7	83.3	51.6	98.8	100	61.9/96.5	78.1	70.7	100
2017 (557)	99.6	95.7	93.6	84.2	53.5	98.7	100	59.8/95.0	78.1	71.6	100
2018 (620)	99.5	97.9	95.8	86.3	53.2	99.5	100	65.0/96.9	80.8	74.4	100
2019 (550)	99.5	98.5	96.2	87.5	51.3	100	100	62.7/97.6	78.7	73.6	100
2020 (467)	98.9	97.4	95.2	86.7	57.4	99.8	100	67.0/96.8	81.6	74.1	100
US Census Division											
New England (343)	99.4	98.8	97.6	83.1	55.4	98.8	100	71.7/98.5	72.0	83.6	100
Middle Atlantic (337)	99.4	97.3	95.7	85.8	57.9	99.1	100	62.6/96.4	77.7	76.2	100
East North Central (516)	100	97.1	94.5	85.1	55.6	99.6	100	65.7/96.5	82.7	77.7	100
West North Central (312)	100	97.1	96.1	90.4	46.2	99.4	100	62.2/96.8	82.1	67.6	100
South Atlantic (283)	98.2	95.8	93.2	88.0	47.0	99.3	100	58.7/94.7	84.5	73.9	100
East South Central (239)	98.7	95.8	93.2	77.4	44.4	100	100	57.7/94.6	73.2	68.6	100
West South Central (323)	99.7	98.1	92.5	82.0	38.7	99.4	100	49.2/96.3	74.6	57.3	100
Mountain (195)	99.0	97.4	94.2	87.7	64.6	99.0	100	68.7/95.9	82.1	70.8	100
Pacific (245)	100	99.2	98.8	91.4	73.9	99.6	100	72.7/98.8	86.1	74.7	100
Phenotypes											
Ceftriaxone-nonsusceptible (72)	79.2	0.0	16.7	22.2	0.0	100	100	0.0/18.1	15.3	4.2	100
Clindamycin-resistant (386)	97.4	85.8	73.8	0.0	0.3	98.7	100	19.4/79.5	9.3	41.7	100
Erythromycin-resistant (1286)	98.8	94.4	89.6	68.9	0.0	99.3	100	34.6/92.5	59.5	54.0	100
Levofloxacin-nonsusceptible (18)	100.0	100	94.1	72.2	44.4	0.0	100	50.0/100	61.1	20.0	100
Oral penicillin-resistant ^b^ (325)	95.4	78.5	58.6	57.8	6.8	98.5	100	0.0/70.5	52.0	28.0	100
Parenteral penicillin-nonsusceptible ^b^ (96)	84.4	38.5	5.2	16.7	0.0	100	100	0.0/0.0	10.4	1.0	100
Tetracycline-resistant (570)	98.2	89.3	80.3	37.0	8.2	98.8	100	23.7/84.9	0.0	40.4	100
SMX-resistant (453)	97.6	85.4	73.0	64.0	17.4	98.2	100	11.3/80.1	55.4	0.0	100
MDR ^c^ (505)	97.0	85.7	76.4	25.7	0.0	98.2	100	14.3/81.0	5.0	29.3	100
Serotypes ^d^											
All (625)	99.7	97.6	94.6	83.2	54.6	98.7	100	64.3/95.8	79.5	76.1	100
35B (75)	100	98.7	94.7	93.3	16.0	100	100	5.3/100	90.7	73.3	100
3 (61)	100	100	100	91.8	90.2	100	100	98.4/100	85.2	98.4	100
11A/11D (50)	100	100	100	92.0	42.0	98.0	100	94.0/100	90.0	91.8	100
23A (38)	100	100	100	68.4	52.6	100	100	31.6/100	73.7	84.2	100
22A/22F (33)	100	100	100	97.0	63.6	87.9	100	97.0/100	97.0	97.0	100
15A/15F (33)	100	100	100	18.2	6.1	97.0	100	18.2/100	15.2	48.5	100
23B (32)	100	100	100	100	68.8	100	100	62.5/100	100	62.5	100
19A (32)	93.8	62.5	18.8	18.8	6.2	96.9	100	12.5/34.4	12.5	12.5	100
16F (30)	100	100	100	100	96.7	100	100	96.7/100	100	96.7	100
19F (30)	100	100	96.7	90.0	83.3	100	100	83.3/96.7	86.7	86.7	100
15B/15C (26)	100	100	100	88.5	42.3	100	100	61.5/100	61.5	53.8	100
Other (185)	100	98.9	98.4	89.7	65.4	99.5	100	79.5/97.8	85.9	76.8	100

^a^ BPR, ceftobiprole; CRO, ceftriaxone; AMC, amoxicillin–clavulanate; CLI, clindamycin; ERY, erythromycin; LEV, levofloxacin; LZD, linezolid; oral/parenteral PEN, penicillin; TET, tetracycline; TMP-SMX, trimethoprim–sulfamethoxazole; VAN, vancomycin. FDA susceptibility breakpoint (≤0.5 mg/L) used for ceftobiprole, and CLSI susceptibility breakpoints applied to other agents, as follows: ceftriaxone, ≤1 mg/L; amoxicillin–clavulanate, ≤2/1 mg/L; clindamycin, ≤0.25 mg/L; erythromycin, ≤0.25 mg/L; levofloxacin, ≤2 mg/L; linezolid, ≤2 mg/L; oral/parenteral penicillin, ≤0.06 mg/L/≤2 mg/L (non-meningitis isolates); tetracycline, ≤1 mg/L; trimethoprim–sulfamethoxazole, ≤0.5 mg/L; vancomycin, ≤1 mg/L. ^b^ Oral penicillin-resistant with MIC ≥ 2 mg/L, isolates nonsusceptible to parenteral penicillin MIC ≥ 4 mg/L are shown instead due to the small number (11) of resistant isolates. ^c^ MDR *S. pneumoniae* isolates were defined as nonsusceptibile to ≥3 of the following antimicrobial agents: parenteral penicillin (MIC, ≥4 mg/L), ceftriaxone (MIC, ≥2 mg/L), erythromycin (MIC, ≥0.5 mg/L), clindamycin (MIC, ≥0.5 mg/L), levofloxacin (MIC, ≥4 mg/L), tetracycline (MIC, ≥2 mg/L), and trimethoprim–sulfamethoxazole (MIC, ≥1 mg/L). ^d^ Susceptibilities against serotypes or serogroups with >25 isolates are described individually. Other less common serotypes or serogroups were represented by ≤22 isolates each and included: 6C/6D (22), 9N/9L (18), 7C/7B/40 (15), 33F/33A/37 (14), 10A (13), 35F/47F (11), 17F (8), 21 (8), 8 (7), 34 (7), 20 (7), 31 (7), 6B (5), 17A (5), 38/25F/25A (4), 23F (4), 12F/12A/44/46 (4), 6A (3), 9V/9A (2), 35D (2), 28A/28F (1), 33B/33D, (1), 4 (1), 7F (1), 14 (1), 6E (1), nontypeable (13).

## Data Availability

The original contributions presented in this study are included in the article. Further inquiries can be directed to the corresponding author.
